# Changes in self-reported alcohol consumption at high and low consumption in the wake of the COVID-19 pandemic: a test of the polarization hypothesis

**DOI:** 10.3389/fpsyt.2025.1516090

**Published:** 2025-09-17

**Authors:** Alexander Tran, Huan Jiang, Shannon Lange, Mindaugas Štelemėkas, Daumantas Stumbrys, Ilona Tamutienė, Jürgen Rehm

**Affiliations:** ^1^ Institute for Mental Health Policy Research, Centre for Addiction and Mental Health, Toronto, ON, Canada; ^2^ Campbell Family Mental Health Research Institute, Centre for Addiction and Mental Health, Toronto, ON, Canada; ^3^ Dalla Lana School of Public Health, University of Toronto, Toronto, ON, Canada; ^4^ Department of Psychiatry, University of Toronto, Toronto, ON, Canada; ^5^ Institute of Medical Science, University of Toronto, Toronto, ON, Canada; ^6^ Health Research Institute, Faculty of Public Health, Lithuanian University of Health Sciences, Kaunas, Lithuania; ^7^ Department of Preventive Medicine, Faculty of Public Health, Lithuanian University of Health Sciences, Kaunas, Lithuania; ^8^ Sociology Department, Vytautas Magnus University, Kaunas, Lithuania; ^9^ Program on Substance Abuse & WHO European Region Collaboration Centre, Public Health Agency of Catalonia, Barcelona, Catalonia, Spain; ^10^ Zentrum für Interdisziplinäre Suchtforschung (ZIS), Universitätsklinikum Hamburg-Eppendorf, Hamburg, Germany

**Keywords:** COVID-19 pandemic, alcohol consumption, drinking polarization, heavy drinking, drinking patterns

## Abstract

**Background:**

The Coronavirus Disease 2019 (COVID-19) pandemic and associated public health measures impacted alcohol use. It was hypothesized that the COVID-19 pandemic led to a polarization of drinking–that is, heavy drinkers increased their drinking, while light to moderate drinkers decreased their drinking. The aim of the current study was to probe deeper into this hypothesis to determine precisely which segment of heavy drinkers increased their consumption.

**Methods:**

We obtained data from the Reducing Alcohol Related Harm Standard European Alcohol Survey for Lithuania, for two separate years; 2015 (n = 1354, mean age = 41.04 ± 13.04, females = 680, 50.2%) and 2020 (n = 1015, mean age = 42.27 ± 13.44, females = 513, 50.5%). Average daily consumption (in grams per day) was decomposed into deciles and compared pre-COVID-19 to onset of the COVID-19 pandemic across the 10^th^, 9^th^, and 1^st^ deciles. To test our hypothesis we conducted a non-parametric pairwise comparison (Mann-Whitney U test) of alcohol consumption at the upper deciles. We also conducted a multivariate linear regression using mental well-being and sociodemographic variables as predictors of consumption.

**Results:**

Alcohol consumption decreased from 2015 to 2020, mean = 11.49 cl of pure alcohol (SD = 8.23) vs. mean = 10.71 cl of pure alcohol (SD = 12.12), *p* <.00001, respectively. However, in the highest decile there was an increase from 2015 to 2020 mean = 29.26 cl of pure alcohol (SD = 5.44) vs. mean = 39.23 cl of pure alcohol (SD = 20.58), *p* = .0003, respectively. This reversal pattern was not observed in the second highest nor the lowest decile. The multivariate model was significant (F(11,1881) = 20.85, p <.00001, adjusted R^2^ = 0.10) and showed significant year by sex interaction (p = .021) and year by occupation interaction (p = .023) on alcohol consumption.

**Conclusion:**

Although COVID-19 was associated with declines in alcohol consumption, in Lithuania it appears that there was an increase in consumption among the heaviest drinkers, driven partially by a smaller difference in consumption between males and females.

## Introduction

During the Coronavirus Disease 2019 (COVID-19) pandemic there was a considerable shift in behavior as people experienced fear, anxiety, and uncertainty around this novel disease (1–3). Across the globe, countries implemented lockdowns to prevent the spread of the disease which, among many consequences, led to widespread social isolation (4–6). An important question regarding the outcomes of the lockdown is how peoples’ health behavior, such as alcohol use, may have been impacted (1).

COVID-19 associated lockdowns and other measures, had a complex effect on alcohol consumption and alcohol-attributable harm. In the beginning of the epidemic, in March 2020, it had been predicted (7) that the effects would be primarily twofold, overall, there would be less availability and affordability of alcohol, leading to a decrease in consumption (for details on these mechanisms see (8). On the other hand, in previous economic and natural crisis situations, alcohol was used as coping mechanism for stress, fear and anxiety as would be the case with a novel pandemic like COVID-19 (see (9, 10) for alcohol as coping mechanism). Together, both mechanisms were predicted to lead to polarization of drinking, meaning that overall there would be a reduction of alcohol consumption, but certain groups, especially groups with prior heavier drinking patterns and/or mental problems, would increase their consumption (11). In support of an overall reduction, previous research on Danish, Swiss, Norwegian, and European survey data has indicated that individuals consumed less alcohol during the early months of the pandemic, as well as a year into the pandemic (12–15). In the first year of COVID-19 alone, surveillance reports by the WHO also found that global consumption indeed went down by 10.3% and in the EU by 6.6% (16).

These findings, however, are across the entire population, meanwhile researchers have found evidence for the polarization of drinking hypothesis (13, 14, 17–19). In fact, a meta-analysis of survey studies probing alcohol consumption in adults found that the proportion of drinkers that reported a decrease in consumption was only slightly larger than the proportion that reported an increase (13). In that study a meta-regression attempted to identify moderators to illuminate whether certain study characteristics affected changes in consumption but none were significant (13). Notably, the authors provided a narrative summary of studies including individuals with alcohol use disorders (AUD) noting that there were significant increases in consumption among that population. Thus, quantitative studies testing for specific moderators leading to increased consumption are warranted.

Some work has explored this nuance, for example one study of a representative survey analyzed trends within sub populations found that only young adults in Switzerland (15–24 years of age) increased consumption (15). Similarly, a study of Italian young adults showed that younger men, and those reporting greater negative experiences during COVID-19 were more likely to score higher on the Alcohol Use Disorders Identification Test (AUDIT) (20). As well, a study in Israel found that young adults (15–18 years of age) increased risky behaviours during the pandemic, including binge drinking (21). Although these findings indicate that some segments of the population increased drinking, they do not directly support the polarization hypothesis per se (e.g., comparison of heavy and non-heavy drinkers). Rossow et al., provided some direct evidence by conducting an analysis of two general population surveys after the onset of the COVID-19 pandemic (12). The authors modelled estimated changes in consumption based on baseline reports of consumption, showing that the upper 5-10% of drinkers increased consumption. Aside from this study others have not tried to precisely identify “the heaviest drinkers” and demonstrate increased drinking in this sub population following the pandemic.

There is evidence of an association between higher consumption and symptoms of anxiety or depression, job insecurity, loneliness or generally lower mental well-being (21–25). However, these studies are largely cross-sectional, indicating that these variables are correlated, but the mechanism (and causal direction) remains uncertain. Generally, it is shown that mental distress (e.g., anxiety and depression) co-occurs with alcohol use disorders but is not necessarily causal (26, 27). As well, there is evidence that job loss was associated with increased drinking (28).

The primary aim of the current study was to probe further into the polarization of drinking hypothesis with a direct test of the change in consumption in a general adult population survey. Using data from Lithuania from two standardized European surveys before and after the onset of the COVID-19 pandemic we investigated the impact of the pandemic on consumption in general, and across deciles. A secondary aim of this study was to test and control for demographic characteristics and mental well-being to identify moderators of the polarization of drinking hypothesis. We also aimed to probe deeper into the polarization hypothesis and analyze characteristics of individuals that increased consumption.

## Method

### Data source

Data were analyzed from the RARHA SEAS (Reducing Alcohol Related Harm Standard European Alcohol Survey), for more information related to the survey and design, see (29). The RARHA SEAS survey was a standardized survey distributed across 33 European countries that measured respondents drinking patterns, drinking preferences, (both variables used to compute estimated daily alcohol consumption) motivations for drinking, general health, and demographic information.

### Survey design and inclusion criteria

From the RARHA SEAS survey, we analyzed data for Lithuania only. In both surveys, the target population ranged in age from 18 to 64 years and the goal of the survey was to generate a representative sample. According to the RARHA SEAS study documentation, “multi-stage stratified probability samples were used. Firstly three strata according to the type of place of residence were distinguished: (1) the biggest cities, (2) regional centers, and (3) rural areas. Then points were selected. In the form of ordinary probability sampling, streets were selected in each sampling point (city/town/rural area)”. Overall there was a high non-response rate, however other research and countries had similar rates (see (30)). The following general inclusion criteria were used according to the RARHA SEAS documentation: a general population survey including information on alcohol consumption and/or alcohol related harm (no targeted populations); nationwide data (no regional surveys); age range: at least 18–64 years.

### Sample characteristics

For the purposes of our study (testing the polarization of drinking hypothesis) we included only individuals that were consuming alcohol. In total there were 2528 responses across both surveys, but excluding non-drinkers resulted in a final sample of 2252. This sample was split into pre-COVID-19 (data collection year 2015, n=1354, females = 680 [50.2%], mean age =41.04 ± 13.04 years) and onset of the COVID-19 pandemic (data collection year, 2020, n=898, females = 438 [48.7%], mean age =42.41 ± 13.17 years). The onset of the COVID-19 pandemic data were collected between August and September 2020. This second survey fell between two lockdowns periods in Lithuania, which were imposed in Spring 2020 and Autumn 2020. The surveys were conducted using computer-assisted face-to-face interviews (CAPI) in the homes of the respondents. The response rate was 38.9% in 2015 and 38.0% in 2020.

### Outcome measures

The survey measured alcohol consumption using multiple beverage-specific alcohol consumption measures (one on beer, wine, and spirits). Individuals were asked to report on frequency and amount for each beverage. Specifically, they were asked “How often did you drink (beer/wine/spirits) in the past 12 months? Would you say that you drank” and were given the options: ‘almost daily’,’weekly’, ‘monthly’, ‘less frequently’ or ‘never’. Based on their response, they gave a more precise measure (e.g., everyday, or 5–6 days a week if they selected daily, or 3–4 days a week or 1–2 days a week, if they selected weekly). This outcome measure was repeated for each alcohol type. Participants were then asked “How much (beer/wine/spirits) did you drink usually on the days when you drank wine in the past 12 months? From the list of units below please select one and report how many units did you usually drink” and participants responded with a specific volume of consumption. The frequency and volume were aggregated into an estimated measure of average daily alcohol consumption in centiliters (cl). Values were capped when computing estimated daily alcohol consumption, with a maximum of 50 cl of pure alcohol per beverage type (about 28 standard drinks, see (30) for full description of variables).

Mental well-being was measured on a single-item rated using a 5-point Likert-type scale with the prompt: “How would you rate your psychological well-being? Is it” (1- Very good to 5 – Very bad).

### Moderator/control variables

We included 3 additional moderator variables in our analysis. Social class was a categorical variable consisting of 4 levels (blue collar worker, white collar worker, professional/manager, or business person). This variable was determined from a collection of other survey items that probed current employment status, previous occupation, whether they were professionally active, or the employment of the head of household (see (30)). Age was a free-response numeric variable and finally, sex was a forced choice item, either male or female.

### Analysis

Using independent samples t-tests, we compared daily alcohol consumption as well as mental well-being from each survey (2015 compared to 2020) separated by the moderator variables. To more precisely test our main hypothesis, we first performed a Kolmogorov-Smirnov (KS) test to determine if the distributions of the alcohol consumption responses differed between the 2015 and the 2020 surveys (31). For non-parametric distributions, we followed this analysis up with a Mann-Whitney U (MWU) test (32) to compare the average values of daily consumption decreased between the years. Next, we separated daily alcohol consumption into deciles to test the polarization hypothesis. To determine the precise boundary of the polarization we performed the same MWU test for the highest (10^th^) and second highest (9^th^) deciles. The results would indicate whether a decile below the heaviest drinkers would also contribute to the polarization effect. As a comparison, we also looked at the lowest decile (1^st^) of consumption.

For our secondary aim we first conducted a multivariate analysis including all moderator variables as well as year and mental well-being in a model predicting alcohol consumption. Thus, in this model we included age, sex, mental well-being, social class (described above) and year, in addition to interaction terms. We interacted year with each predictor variable in a stepwise manner, to determine if the change in consumption between years was moderated by any relevant demographic variable. Finally, only significant interactions were added to a final model.

As well, we conducted a sensitivity analysis with all the same tests using a natural log-transformed alcohol consumption variable (see **Supplementary Analyses**). All analyses were conducted in R Version 4.1.5 (33).

## Results

**Figure 1:**
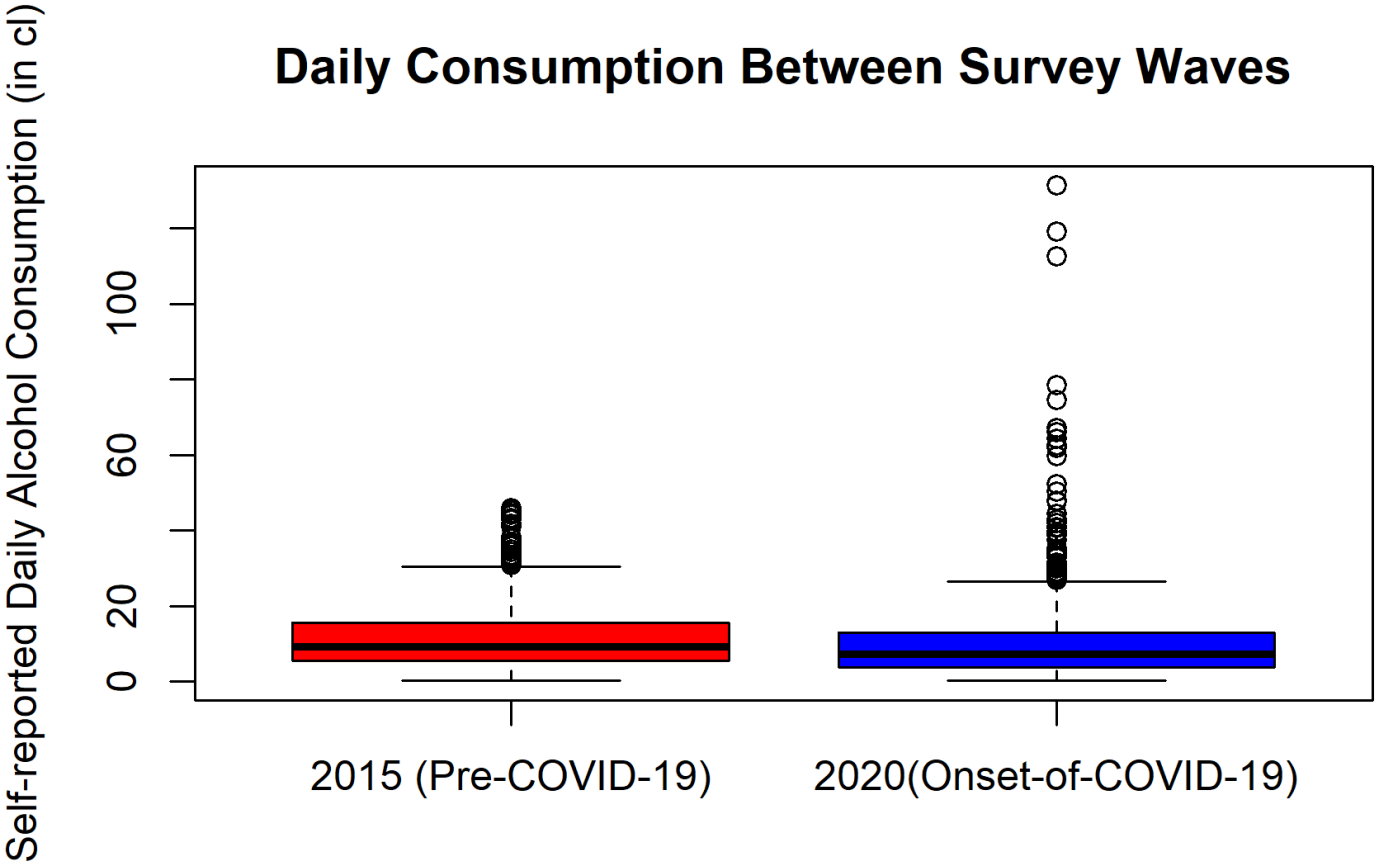
A distribution of values for daily alcohol consumption (in cl) pre and onset of the COVID-19 pandemic as a Box-and-Whisker plot, where boxes represent the interquartile range (25^th^, median, and 75^th^ percentiles) and outliers (scatterplot).

The results of the comparison between years for alcohol consumption and mental well-being across demographic variables can be seen in **Table 1**. Notably, males decreased consumption from 2015 to 2020 (t (654.77) = 2.09, p = .037) as did white collar workers (t (254.29) = 2.62, p = .009). There were no differences in mental well-being from 2015 to 2020 across any demographic variable. In our more focused analyses, the KS test determined that the two surveys had significantly different distributions (D = .14, p <.00001, see **Figure 1**). The MWU test showed that across the whole population there was significantly higher alcohol consumption in 2015 than in 2020 (p <.00001, mean consumption = 11.49 ± 8.23 cl of pure alcohol vs. mean consumption = 10.71 ± 12.12 cl of pure alcohol, respectively) equating to roughly 6.5 vs. 6 standard drinks per day (1 standard drink = 14 grams of pure alcohol), respectively.

**Table 1:** Analysis of change in alcohol consumption and well-being between pre-and onset of the COVID-19 pandemic (means, and SD) across various demographic categories.

	Year				
	2015		2020		p-value
Alcohol consumption	Sample	Mean (SD)	Sample	Mean (SD)	
Sex					
Males	n = 663	14.77 (9.25)	n = 419	13.17 (13.82)	p = .037*
Females	n = 650	8.16 (5.27)	n = 404	8.16 (9.42)	*p = .99*
Social class					
Blue collar	n =695	11.95 (8.34)	n = 266	11.02 (10.88)	*p = .21*
White collar	n = 129	12.7 (9.19)	n = 128	9.79 (8.64)	p = .0093*
Manager/Professional	n = 414	10.51 (7.81)	n = 171	11.27 (15.81)	*p = .55*
Business owner	n = 58	11.39 (7.82)	n = 32	10.08 (9.9)	*p = .53*
Not reported (NA)	n = 17	8.61 (4.82)	n = 226	10.54 (12.32)	*p = .18*
Age (dichotomized)					
Less than 40	n = 587	10.82 (7.88)	n = 351	10.21 (11.38)	*p = .37*
40 and older	n = 726	12.04 (8.47)	n = 472	11.09 (12.64)	*p = .15*
Mental well-being	Sample	Mean (SD)	Sample	Mean (SD)	p-value
Sex					
Males	n = 663	2.21 (0.8)	n = 419	2.2 (0.86)	*p = .78*
Females	n = 650	2.15 (0.76)	n = 404	2.24 (0.84)	*p = .085†*
Social class					
Blue collar	n = 695	2.19 (0.76)	n = 266	2.19 (0.79)	*p = .99*
White collar	n = 129	2.09 (0.78)	n = 128	2.12 (0.83)	*p = .81*
Manager/Professional	n = 414	2.21 (0.81)	n = 171	2.08 (0.74)	*p = .069†*
Business owner	n = 58	2.1 (0.74)	n = 32	2.22 (0.71)	*p = .46*
Not reported (NA)	n = 17	2.24 (0.83)	n = 226	2.41 (0.98)	*p = .41*
Age (dichotomized)					
Less than 40	n = 587	2.09 (0.77)	n = 351	2.11 (0.9)	*p = .75*
40 and older	n = 726	2.26 (0.78)	n = 472	2.3 (0.8)	*p = .35*

SD, standard deviation; * *p* <.05, *† p* <.1.

**Figure 2:**
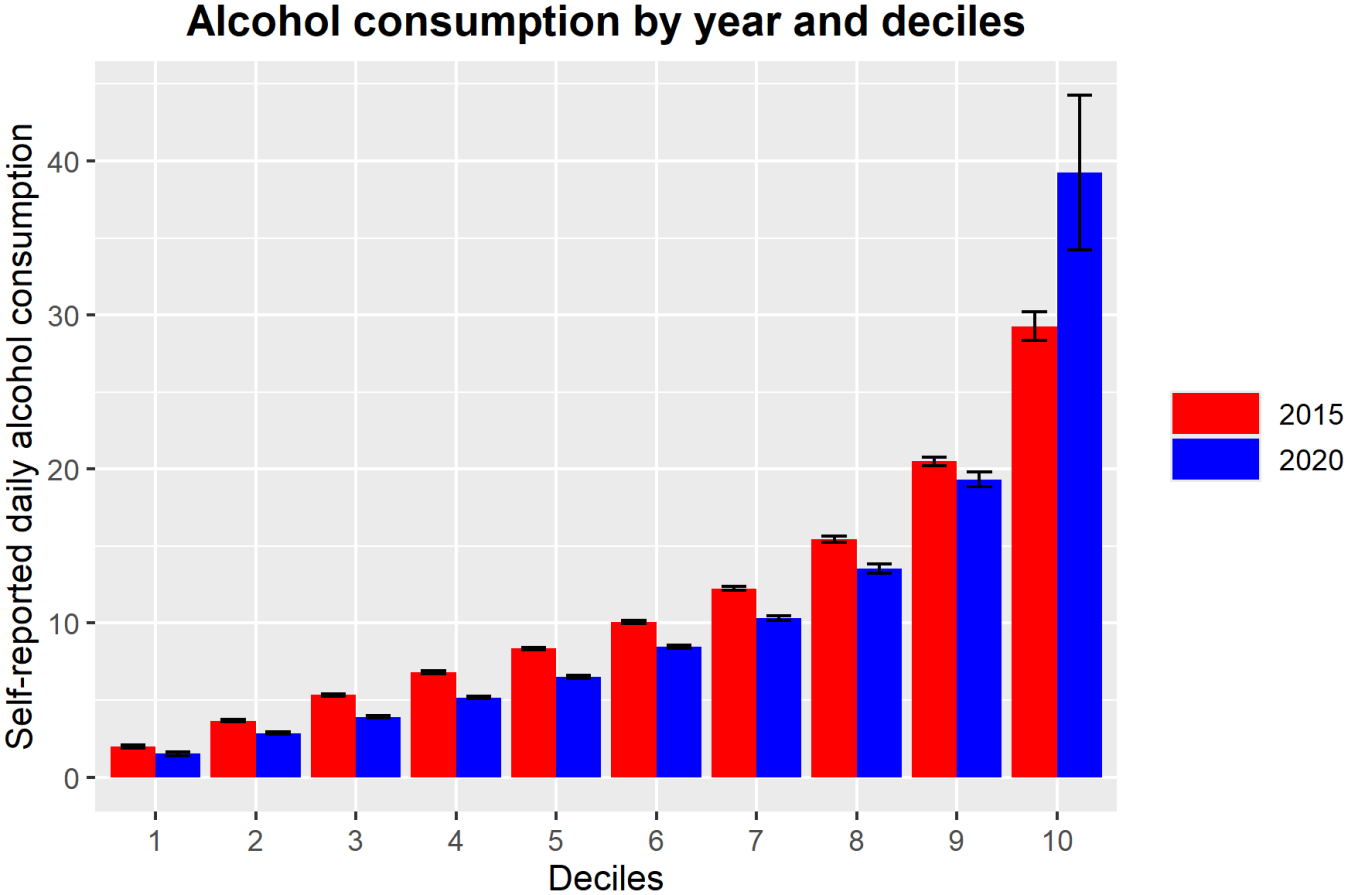
Bar graph of average estimated daily alcohol consumption separated by deciles comparing pre-pandemic (2015) in red and onset of the COVID-19 pandemic (2020) in blue. Bars represent standard error.

The data were then split into deciles of daily alcohol consumption (see **Figure 2**). We first conducted a KS-test on only those in the 10^th^ decile (heaviest drinkers). The KS test showed significantly different distributions (D = .30, p = .0002), and thus we followed up with a MWU test which showed a significant increase in daily consumption in the heaviest drinkers after the onset of the COVID-19 pandemic (p = .0003, mean consumption = 29.26 ± 5.44 cl of pure alcohol vs. mean consumption = 39.23 ± 20.58 cl of pure alcohol, respectively) equating to roughly 16.5 vs. 22 standard drinks per day, before and after the onset of the COVID-19 pandemic respectively. The tests were repeated for the second highest decile (9^th^), and the lowest decile (1^st^), and the results indicated that unlike the 10^th^ decile, there was significantly higher alcohol consumption before the pandemic, than after the onset of the COVID-19 pandemic (see **Table 2**).

**Table 2:** Analysis of change in alcohol consumption pre-and onset of the COVID-19 pandemic, means, and SD of consumption for the 1st and 9th deciles.

Decile	Test type	Statistic	p-value	Consumption in cl 2015 M (SD)	Consumption in cl 2020 M (SD)
Decile 1	KS test,	D = .41,	*p* <.0001	1.98 (0.66)	1.51 (0.51)
	MWU test	W = 7365	*p* <.0001		
Decile 9	KS test,	D = .32,	*p* <.0001	20.49 (1.70)	19.33 (2.25)
	MWU test	W = 7299	*p* <.0001		

KS, Kolmogorov-Smirnov; MWU, Mann-Whitney U; M, mean; SD, standard deviation; cl, centiliters.

Next, we conducted our multivariate analysis to further understand subsections of the population that consumed more alcohol. The separated linear regression and interaction terms can be found in the **Supplementary Analyses** (See **Supplementary Tables S1**-**S4**). There was a significant interaction of year by sex (t(1881) = 2.27, p = .023) and year by occupation (t(1881) = 2.3, p = .021, see **Table 3**). We decomposed this interaction and found that in 2015, males had higher consumption as compared to females (t(1289) = -15.98, p <.0001) but the effect size was weaker in 2020 ((t(590) = -4.46, p <.0001). As well, in 2015, managers and professionals consumed less alcohol than blue collar workers (t(1289) = -2.60, p = .009), but there was no difference in consumption between these occupations in 2020 (t(590) = 1.01, p = .3).

**Table 3:** General linear model including all predictor variables with interactions between social class and gender by year.

Predictor	Estimate	Standard Error	t value	Pr(>|t|)	
(Intercept)	1,853.482	582.714	3.181	0.0015	**
Age	0.048	0.017	2.901	0.0038	**
Mental well-being	0.223	0.270	0.826	0.4090	
Year	-0.910	0.289	-3.150	0.0017	**
Sex	-848.046	370.228	-2.291	0.0221	*
White collar worker	554.165	533.552	1.039	0.2991	
Manager/Professional	-998.424	432.887	-2.306	0.0212	*
Business person	-48.166	852.204	-0.057	0.9549	
Year * Sex	0.418	0.184	2.274	0.0231	*
Year * White collar worker	-0.275	0.265	-1.038	0.2995	
Year * Manager/Professional	0.495	0.215	2.305	0.0213	*
Year * Business person	0.023	0.423	0.056	0.9557	

Social class uses blue collar worker as comparison category, all terms are relative to blue collar worker daily alcohol consumption.

Signif. codes: 0 <= ‘***’ < 0.001 < ‘**’ < 0.01 < ‘*’ < 0.05.

**Figure 3:**
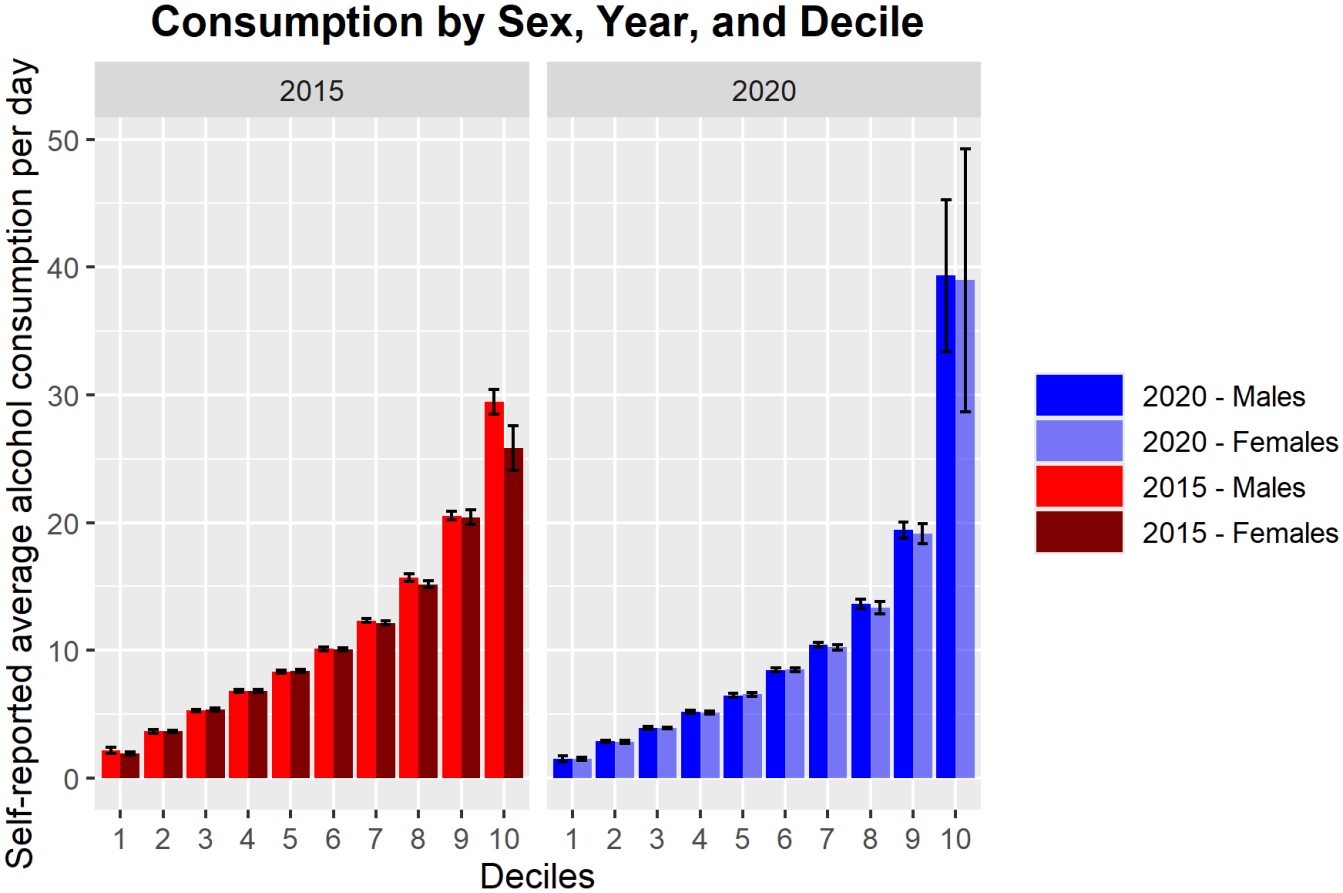
Bar graph of estimated daily alcohol consumption separated by deciles and stratified by sex. The pre-pandemic (left pane, red = males, maroon = females) and onset of the COVID-19 pandemic (right pane, blue = males, light blue = females). Bars represent standard error.

Finally, we conducted an exploratory analysis of the data separated by sex, comparing consumption between years in the 10^th^ decile. The results showed that males consumed more alcohol than females in upper decile in 2015 (KS test, D = .40, p <.0001, MWU test, p <.0001, males mean consumption = 29.45 ± 5.51 vs. females mean consumption = 25.82 ± 1.86), however, in 2020, there was no significant difference between sexes (KS test, D = .15, p = .38, t(75) = .24, p = .81, males mean consumption = 39.33 ± 21.75 cl of pure alcohol vs. females mean consumption = 38.95 ± 21.41 cl of pure alcohol, see **Figure 3**).

## Discussion

Understanding the effect of global crises like the COVID-19 pandemic on alcohol consumption is vital to protecting public health. In the present study using two waves of general population survey data in Lithuania, we analyzed changes in alcohol consumption across varying levels of consumption and identified key demographic characteristics that may have impacted these changes. In general, there was higher self-reported alcohol consumption in 2015 than in 2020, which is in line with some previous research on the effect of COVID-19 on alcohol consumption in Europe (13, 17). Globally, as found by the WHO status report on alcohol consumption, alcohol consumption per capita dropped during the pandemic (16) In on specific European study, general population data was used (the European Alcohol Use and COVID-19 Survey) from 9 European countries, and the authors showed that individuals reported drinking “less” in the past month (as compared the past 12 months) across 3 items on the AUDIT-C (12, 34). These findings were then quantified into estimates of changes in consumption which demonstrated that those above the 90^th^ percentile of consumption were more likely to report increased consumption (12). Our study differs from this study, which was conducted during the pandemic (April 2020-July 2020), and instead we included survey data from well before the pandemic. We found nearly identical effects, such that only the top decile (upper 10%) showed an increase in self-reported consumption, meanwhile the 9^th^ decile showed a decrease in consumption. Thus, like others, we showed that the COVID-19 pandemic had a complex effect on the availability and affordability of alcohol, two key factors in consumption (8). Despite a reduced availability of alcohol due to social distancing measures, and reduced affordability due to an economic crisis, vulnerable segments of the population (heavy drinkers) increased their consumption.

The mechanism of why polarization is a multi-faced and complex issue, however in the present study we attempted to identify key moderators and provide additional quantitative evidence expanding on the polarization of drinking effect. Firstly, when separated by sex, we found that on average males tended to reduce consumption from 2015 to 2020, while females stayed the same. Other studies analyzing consumption during the pandemic have instead found that females were more likely to report higher consumption (18, 35). For instance, a systematic review of studies analyzing consumption found that within Europe there was a consistent in increase in consumption in females (18). A study of mental health and COVID-19 showed that women were more likely to report an increase in consumption along with higher levels of mental distress (36). Secondly, we found that certain professions appeared to consume more alcohol (managers and professionals) which supports another study of Australian women (37). There, the authors found that new/emerging middle-class women (categorized based on household income, social capital, and cultural capital) were the most likely to harbor feelings of anxiety or depression and subsequently report higher alcohol consumption. Contrary to other work, we did not find that younger individuals (younger than 40) increased consumption. Although others have found that young adults tended to increase consumption, we did not find that was the case in our univariate analyses, nor the multivariate model (21, 25). Interestingly, our multivariate analysis showed that mental health was not a significant predictor of consumption. Nor was there a significant interaction of year and mental well-being on consumption. This result is at odds with other research on COVID-19 and mental distress (38). Research on mental health and alcohol consumption generally shows a negative correlation (i.e., worse mental health is associated with more consumption) (36). A possible explanation for our puzzling findings could be the poor reliability and validity of a single Likert-type item mental health. Indeed other work studying the association of mental health and alcohol consumption has used 21-item scales (36) or 5-item (39) scales of anxiety and depression.

In our exploratory analyses we separated the effect at the highest decile by sex to probe further into the effect among heaviest drinkers. We found that although both sexes in the 10^th^ decile increased consumption, females appeared to dramatically increase consumption, resulting in no significant differences in consumption between males and females. This is a stark finding, given that in general males consume more than females and have higher prevalence of alcohol use disorders (40). Interestingly, as shown in the Figure X, across all other deciles, the consumption between sexes was comparable—possibly reflecting the high per capita consumption in Lithuania. Noteworthy is that the sample is relatively small at the upper deciles (only 135 in 2015 and 73 in 2020). Such a small sample resulted in low power for additional analyses beyond sex stratifications.

Overall, despite the dramatic changes during the COVID-19 pandemic, there remained a significant burden of alcohol consumption on the health of heavy drinkers in Lithuania. Importantly, the high level of consumption was seen in a non-negligible segment of the population (10% of the total population).

### Limitations and future directions

Still, there are some limitations of the present study. Although many characteristics of Lithuania lend itself to being a comparable example to other EU and Western countries, it is unique in that there is a high overall level of alcohol consumption, and a high gap in life expectancy between gender (41). Thus, the upper decile of the population, on average, may have a higher level of consumption than in other countries. Second, the analysis was conducted on a general survey that involved two waves of different participants. Thus, although we infer that consumption increased amongst the upper deciles, it is merely speculative (stronger support would come from a direct measure of consumption such as sales data, clinical data, or mortality rates). Third, the response rate in our surveys was quite low (38.9% and 38.0%), which could lead to self-reported alcohol consumption that are biased by selective non-response (29). Finally, as with all self-reported measures, there is some margin of error with respect to response biases (e.g., social desirability bias).

The COVID-19 pandemic was associated with dramatic changes to factors affecting alcohol consumption— from a public policy perspective and from a mental health perspective. Although on a whole, there was a decrease in alcohol consumption, certain segments of the population were affected adversely. We demonstrate, using Lithuania as an example, that vulnerable populations (heavy drinkers, at the upper decile of consumption) had significant increases in consumption which is in line with hypotheses of previous researchers. This finding emphasizes the need to monitor and protect those prone to high alcohol consumption during times of crisis.

## Data Availability

Data can be sent upon reasonable request but are not publicly available due to privacy and confidentiality issues. Requests to access these datasets should be directed to AT, 9trana@gmail.com.

## Supplementary material

Supplementary Table 1 Multivariate model testing the interaction of year and age, daily consumption untransformed.

Supplementary Table 2 Multivariate model testing the interaction of year and mental well-being, daily consumption untransformed.

Supplementary Table 3 Multivariate model testing the interaction of year and social class, daily consumption untransformed.

Supplementary Table 4 Multivariate model testing the interaction of year and sex, daily consumption untransformed.

Supplementary Table 5 Multivariate model testing the interaction of year and age, daily consumption log transformed.

Supplementary Table 6 Multivariate model testing the interaction of year and mental well-being, daily consumption log transformed.

Supplementary Table 7 Multivariate model testing the interaction of year and social class, daily consumption log transformed.

Supplementary Table 8 Multivariate model testing the interaction of year and sex, daily consumption log transformed.

Supplementary Table 9 Final multivariate model with interactions, daily consumption log transformed.

Supplementary Table 10 Correlation of well-being and consumption by year.
