# Supplementary material for: Changes in self-reported alcohol consumption at high and low consumption in the wake of the COVID-19 pandemic: a test of the polarization hypothesis
Source: Front Psychiatry. 2025 Sep 17;16:1516090. doi: 10.3389/fpsyt.2025.1516090 (PMC12484121; doi:10.3389/fpsyt.2025.1516090)
Supplement: Supplementary Table 1 — Multivariate model testing the interaction of year and age, daily consumption untransformed. [file Table1.docx]

# Supplementary Analyses

Table S1. Multivariate model testing the interaction of year and age, daily consumption untransformed

|  | Estimate | Standard Error | t value | Pr(>\|t\|) |  |
| --- | --- | --- | --- | --- | --- |
| (Intercept) | -140.629 | 643.557 | -0.219 | 0.8270 |  |
| Sex | -5.985 | 0.424 | -14.123 | 0.0000 | *** |
| White collar worker | 0.326 | 0.652 | 0.501 | 0.6168 |  |
| Manager/Professional | -0.418 | 0.483 | -0.866 | 0.3869 |  |
| Business person | -0.782 | 1.009 | -0.775 | 0.4384 |  |
| Mental well-being | 0.214 | 0.271 | 0.792 | 0.4285 |  |
| Year | 0.079 | 0.319 | 0.246 | 0.8054 |  |
| Age | 12.782 | 14.817 | 0.863 | 0.3884 |  |
| Year * Age | -0.006 | 0.007 | -0.859 | 0.3902 |  |
| *Signif. codes: 0 <= '***' < 0.001 < '**' < 0.01 < '*' < 0.05* | | | | | |

Table S2. Multivariate model testing the interaction of year and mental well-being, daily consumption untransformed

|  | Estimate | Standard Error | t value | Pr(>\|t\|) |  |
| --- | --- | --- | --- | --- | --- |
| (Intercept) | -511.020 | 537.438 | -0.951 | 0.3418 |  |
| Sex | -5.967 | 0.424 | -14.085 | 0.0000 | *** |
| White collar worker | 0.320 | 0.652 | 0.491 | 0.6234 |  |
| Manager/Professional | -0.433 | 0.482 | -0.898 | 0.3693 |  |
| Business person | -0.751 | 1.009 | -0.745 | 0.4566 |  |
| Age | 0.047 | 0.017 | 2.808 | 0.0050 | ** |
| Year | 0.262 | 0.267 | 0.984 | 0.3252 |  |
| Mental well-being | 417.775 | 234.097 | 1.785 | 0.0745 | . |
| Year * Mental well-being | -0.207 | 0.116 | -1.784 | 0.0746 | . |
| *Signif. codes: 0 <= '***' < 0.001 < '**' < 0.01 < '*' < 0.05* | | | | | |

Table S3. Multivariate model testing the interaction of year and social class, daily consumption untransformed

|  | Estimate | Standard Error | t value | Pr(>\|t\|) |  |
| --- | --- | --- | --- | --- | --- |
| (Intercept) | 674.134 | 266.091 | 2.533 | 0.0114 | * |
| Sex | -6.052 | 0.424 | -14.275 | 0.0000 | *** |
| Age | 0.049 | 0.017 | 2.943 | 0.0033 | ** |
| Mental well-being | 0.247 | 0.270 | 0.913 | 0.3611 |  |
| Year | -0.325 | 0.132 | -2.466 | 0.0137 | * |
| White collar worker | 425.000 | 531.109 | 0.800 | 0.4237 |  |
| Manager/Professional | -1,148.017 | 428.335 | -2.680 | 0.0074 | ** |
| Business person | -80.772 | 853.027 | -0.095 | 0.9246 |  |
| Year * White collar worker | -0.210 | 0.263 | -0.799 | 0.4243 |  |
| Year * Manager/Professional | 0.569 | 0.212 | 2.679 | 0.0074 | ** |
| Year * Business person | 0.040 | 0.423 | 0.094 | 0.9252 |  |
| *Signif. codes: 0 <= '***' < 0.001 < '**' < 0.01 < '*' < 0.05* | | | | | |

Table S4. Multivariate model testing the interaction of year and sex, daily consumption untransformed

|  | Estimate | Standard Error | t value | Pr(>\|t\|) |  |
| --- | --- | --- | --- | --- | --- |
| (Intercept) | 1,768.409 | 572.462 | 3.089 | 0.0020 | ** |
| Age | 0.047 | 0.017 | 2.809 | 0.0050 | ** |
| White collar worker | 0.220 | 0.652 | 0.337 | 0.7359 |  |
| Manager/Professional | -0.479 | 0.483 | -0.992 | 0.3215 |  |
| Business person | -0.817 | 1.008 | -0.811 | 0.4176 |  |
| Mental well-being | 0.194 | 0.271 | 0.718 | 0.4731 |  |
| Year | -0.868 | 0.284 | -3.058 | 0.0023 | ** |
| Sex | -936.921 | 365.746 | -2.562 | 0.0105 | * |
| Year * Sex | 0.462 | 0.181 | 2.545 | 0.0110 | * |
| *Signif. codes: 0 <= '***' < 0.001 < '**' < 0.01 < '*' < 0.05* | | | | | |

Table S5. Multivariate model testing the interaction of year and age, daily consumption log transformed

|  | Estimate | Standard Error | t value | Pr(>\|t\|) |  |
| --- | --- | --- | --- | --- | --- |
| (Intercept) | 7.565 | 54.260 | 0.139 | 0.8891 |  |
| Sex | -0.554 | 0.036 | -15.512 | 0.0000 | *** |
| White collar worker | 0.042 | 0.055 | 0.772 | 0.4401 |  |
| Manager/Professional | -0.069 | 0.041 | -1.693 | 0.0906 | . |
| Business person | -0.088 | 0.085 | -1.036 | 0.3005 |  |
| Mental well-being | 0.009 | 0.023 | 0.405 | 0.6858 |  |
| Year | -0.002 | 0.027 | -0.089 | 0.9294 |  |
| Age | 1.854 | 1.249 | 1.484 | 0.1379 |  |
| Year * Age | -0.001 | 0.001 | -1.481 | 0.1389 |  |
| *Signif. codes: 0 <= '***' < 0.001 < '**' < 0.01 < '*' < 0.05* | | | | | |

Table S6. Multivariate model testing the interaction of year and mental well-being, daily consumption log transformed

|  | Estimate | Standard Error | t value | Pr(>\|t\|) |  |
| --- | --- | --- | --- | --- | --- |
| (Intercept) | 34.626 | 45.352 | 0.764 | 0.4453 |  |
| Sex | -0.554 | 0.036 | -15.502 | 0.0000 | *** |
| White collar worker | 0.042 | 0.055 | 0.762 | 0.4460 |  |
| Manager/Professional | -0.069 | 0.041 | -1.686 | 0.0921 | . |
| Business person | -0.086 | 0.085 | -1.013 | 0.3112 |  |
| Age | 0.004 | 0.001 | 3.192 | 0.0014 | ** |
| year | -0.016 | 0.022 | -0.703 | 0.4822 |  |
| Mental well-being | 23.151 | 19.754 | 1.172 | 0.2414 |  |
| Year * Mental well-being | -0.011 | 0.010 | -1.171 | 0.2416 |  |
| *Signif. codes: 0 <= '***' < 0.001 < '**' < 0.01 < '*' < 0.05* | | | | | |

Table S7. Multivariate model testing the interaction of year and social class, daily consumption log transformed

|  | Estimate | Standard Error | t value | Pr(>\|t\|) |  |
| --- | --- | --- | --- | --- | --- |
| (Intercept) | 111.668 | 22.451 | 4.974 | 0.0000 | *** |
| Sex | -0.561 | 0.036 | -15.676 | 0.0000 | *** |
| Age | 0.005 | 0.001 | 3.312 | 0.0009 | *** |
| Mental well-being | 0.012 | 0.023 | 0.518 | 0.6044 |  |
| year | -0.054 | 0.011 | -4.851 | 0.0000 | *** |
| White collar worker | 24.089 | 44.812 | 0.538 | 0.5909 |  |
| Manager/Professional | -94.757 | 36.140 | -2.622 | 0.0088 | ** |
| Business person | -42.600 | 71.973 | -0.592 | 0.5540 |  |
| Year * White collar worker | -0.012 | 0.022 | -0.536 | 0.5919 |  |
| Year * Manager/Professional | 0.047 | 0.018 | 2.620 | 0.0089 | ** |
| Year * Business person | 0.021 | 0.036 | 0.591 | 0.5548 |  |
| *Signif. codes: 0 <= '***' < 0.001 < '**' < 0.01 < '*' < 0.05* | | | | | |

Table S8. Multivariate model testing the interaction of year and sex, daily consumption log transformed

|  | Estimate | Standard Error | t value | Pr(>\|t\|) |  |
| --- | --- | --- | --- | --- | --- |
| (Intercept) | 202.951 | 48.282 | 4.203 | 0.0000 | *** |
| Age | 0.004 | 0.001 | 3.182 | 0.0015 | ** |
| White collar worker | 0.033 | 0.055 | 0.603 | 0.5463 |  |
| Manager/Professional | -0.073 | 0.041 | -1.803 | 0.0716 | . |
| Business person | -0.091 | 0.085 | -1.071 | 0.2845 |  |
| Mental well-being | 0.008 | 0.023 | 0.337 | 0.7362 |  |
| Year | -0.099 | 0.024 | -4.147 | 0.0000 | *** |
| Sex | -80.494 | 30.847 | -2.609 | 0.0091 | ** |
| Year * Sex | 0.040 | 0.015 | 2.591 | 0.0096 | ** |
| *Signif. codes: 0 <= '***' < 0.001 < '**' < 0.01 < '*' < 0.05* | | | | | |

Table S9. Final multivariate model with interactions, daily consumption log transformed

|  | Estimate | Standard Error | t value | Pr(>\|t\|) |  |
| --- | --- | --- | --- | --- | --- |
| (Intercept) | 213.194 | 49.163 | 4.336 | 0.0000 | *** |
| Age | 0.005 | 0.001 | 3.270 | 0.0011 | ** |
| Mental well-being | 0.010 | 0.023 | 0.429 | 0.6681 |  |
| Year | -0.104 | 0.024 | -4.281 | 0.0000 | *** |
| Sex | -73.045 | 31.236 | -2.338 | 0.0195 | * |
| White collar worker | 35.209 | 45.015 | 0.782 | 0.4342 |  |
| Manager/Professional | -81.879 | 36.522 | -2.242 | 0.0251 | * |
| Business person | -39.793 | 71.899 | -0.553 | 0.5800 |  |
| Year * Sex | 0.036 | 0.015 | 2.321 | 0.0204 | * |
| Year * White collar worker | -0.017 | 0.022 | -0.781 | 0.4349 |  |
| Year * Manager/Professional | 0.041 | 0.018 | 2.240 | 0.0252 | * |
| Year * Business person | 0.020 | 0.036 | 0.552 | 0.5808 |  |
| *Signif. codes: 0 <= '***' < 0.001 < '**' < 0.01 < '*' < 0.05* | | | | | |

Table S10. Correlation of well-being and consumption by year

| Year | Correlation (r) | p-value | 95% CI (Lower) | 95% CI (Upper) | Sample Size (n) | t Statistic | Degrees of Freedom (df) |
| --- | --- | --- | --- | --- | --- | --- | --- |
| 2015 | 0.070 | 0.011 | 0.016 | 0.123 | 1,312 | 2.532 | 1,311 |
| 2020 | 0.014 | 0.686 | -0.054 | 0.082 | 822 | 0.404 | 821 |
